# Intratumoral heterogeneity and chemotherapy-induced alteration of CLDN18.2 expression in resectable gastric cancer

**DOI:** 10.1007/s10147-026-02972-w

**Published:** 2026-01-28

**Authors:** Shinnosuke Nagano, Yukinori Kurokawa, Takaomi Hagi, Yuichi Motoyama, Takuro Saito, Tsuyoshi Takahashi, Kota Momose, Kotaro Yamashita, Koji Tanaka, Tomoki Makino, Kiyokazu Nakajima, Eiichi Morii, Hidetoshi Eguchi, Yuichiro Doki

**Affiliations:** 1 Department of Gastroenterological Surgery, The University of Osaka Graduate School of Medicine, 2-2, Yamadaoka, Suita-Shi, Osaka 565-0871 Japan; 2 Department of Pathology, The University of Osaka Graduate School of Medicine, 2-2, Yamadaoka, Suita-Shi, Osaka 565-0871 Japan

**Keywords:** Gastric cancer, Claudin-18, Intratumoral heterogeneity, Molecular targeted therapy, Immunohistochemistry

## Abstract

**Background:**

Claudin-18 isoform-2 (CLDN18.2) is a novel biomarker and therapeutic target for gastric cancer (GC). It may exhibit the intratumoral heterogeneity and varying expressions between biopsy and surgically resected specimens as well as pre- and post-chemotherapy, which could impact patient selection for the targeted agents.

**Methods:**

CLDN18.2 expression was immunohistochemically evaluated in pretreatment biopsy and surgically resected specimens from 183 patients with pT2-T4 GC who underwent upfront gastrectomy. The intratumoral heterogeneity was evaluated by classifying the distribution of CLDN18.2 positive cells as superficial, invasive-front, or random pattern. Furthermore, a separate cohort of 38 patients who underwent neoadjuvant chemotherapy without zolbetuximab were analyzed to compare the pre- and post-treatment CLDN18.2 status.

**Results:**

CLDN18.2 positivity was observed in 31% (56/183) of patients. Among the 93 patients with 2 + /3 + expression in ≥ 10% of the tumor cells, 81 (87%) had heterogeneous expression patterns, including superficial (n = 46), random (n = 24), and invasive-front (n = 11) patterns. The overall biopsy–surgery concordance rate was 86% (157/183), but it decreased to 73% (59/81) in patients with a heterogeneous expression pattern. Notably, the concordance rate was particularly low in the superficial pattern, at only 65% (30/46). Among the 38 patients who underwent neoadjuvant chemotherapy, only 4 of 11 initially CLDN18.2-positive cases remained positive after treatment, although the overall concordance rate was 82% (31/38).

**Conclusion:**

The CLDN18.2 expression demonstrated an acceptable concordance between biopsy and surgically resected specimens. However, high prevalence of heterogeneous expression and tendency for CLDN18.2 positivity to shift to negativity following chemotherapy existed.

**Supplementary Information:**

The online version contains supplementary material available at 10.1007/s10147-026-02972-w.

## Introduction

Gastric cancer (GC) is a major health issue and is one of the leading causes of cancer death worldwide [1, 2]. In Japan, it has the highest mortality rate among gastroenterological malignancies [3]. At present, the standard of care for resectable GC is surgical resection; however, recurrence following surgery or primarily unresectable GC is often encountered [4]. However, despite recent advances in the development of new agents, the prognosis remains poor [5]. To improve the outcomes of patients with advanced GC, the development of new target agents is urgently needed.

Zolbetuximab, an antibody against isoform 2 of claudin-18 (CLDN18.2), has been recently developed as a novel therapeutic agent for patients with advanced GC. CLDN18.2 is predominant in normal stomach mucosa and remains during malignant transformation in GC [6, 7]. CLDN18.2 was exposed on the surface of tumor cells as tight junctions become disrupted in GC malignant transformation and loss of cell polarity [8, 9]. Two recent phase 3 trials showed the survival benefit of zolbetuximab addition to the standard regimen as a first-line chemotherapy for patients with unresectable or metastatic CLDN18.2-positive GC [10, 11].

The potential concern for CLDN18.2 is its intratumoral heterogeneity, similar to issues encountered in human epidermal growth factor receptor 2 (HER2) immunohistochemistry (IHC) [12, 13]. At present, biopsy specimens are often used to conduct CLDN18.2 testing for unresectable or metastatic GC. However, intratumoral heterogeneity may compromise diagnostic accuracy, particularly when assessment relies solely on biopsy specimens. Despite its clinical relevance, CLDN18.2 heterogeneity remains insufficiently characterized, and no prior study has compared expression between matched biopsy and surgically resected specimens in resectable GC. Understanding this discordance is important, as it may influence patient selection for CLDN18.2-targeted therapies. Another clinical issue, as indicated in the HER2 testing [14–16], is the potential for CLDN18.2 expression to change pre- and post-chemotherapy. With the increasing use of neoadjuvant chemotherapy for resectable GC, sufficient understanding of these features is crucial for selecting appropriate samples and timing for companion diagnostic testing.

In this study, the intratumoral heterogeneity of CLDN18.2 in surgically resected specimens was assessed, and its impact on biopsy specimen evaluation was investigated by comparing the CLDN18.2 expressions between matched biopsy and surgically resected specimens. In addition, the changes in CLDN18.2 expression pre- and post-neoadjuvant chemotherapy without zolbetuximab were examined.

## Patients and Methods

### Patient population and samples

This study included 183 patients with pathological T2–T4 GC who underwent upfront gastrectomy without neoadjuvant chemotherapy and 38 who underwent neoadjuvant chemotherapy followed by gastrectomy at Osaka University Hospital between January 2013 and December 2019. The regimens of neoadjuvant chemotherapy were either S-1 plus oxaliplatin (SOX) or docetaxel, oxaliplatin, and S-1 (DOS), and cases with a histological response of Grade 3 were excluded from the eligibility. Surgically resected formalin-fixed paraffin-embedded (FFPE) specimens were examined for CLDN18.2 expression. All 221 patients were available for the evaluation of CLDN18.2 expression using pretreatment biopsy and surgically resected specimens. The pathological TNM staging was based on the Japanese Classification of Gastric Carcinoma, 15th edition [17]. The study was approved by the Ethical Review Board of the University of Osaka Hospital (No. 8226–10, 24,425). Informed consent to be included in the study was obtained from all patients, in compliance with the Declaration of Helsinki.

### IHC and its evaluations

IHC for CLDN18 was performed on 4-µm FFPE sections. After deparaffinization with xylene and rehydration through graded ethanol, endogenous peroxidase activity was blocked using 0.3% hydrogen peroxide. Antigen retrieval was then performed by autoclaving the slides in citrate buffer (pH 6.0) at 110 °C for 15 min. Slides were incubated overnight at 4 °C with a CLDN18.2 primary antibody (Clone 43-14A, Roche Ventana, Oro Valley, AZ), followed by a 30 min incubation with an anti-mouse secondary antibody (Histofine Simple Stain MAX-PO, Nichirei, Tokyo, Japan) at room temperature. Staining was visualized using 3,3ʹ-diaminobenzidine tetrahydrochloride (DAB Tablet, FUJIFILM Wako Pure Chemical Corporation, Osaka, Japan) for 3 min. To evaluate CLDN18.2, the intensity of tumor cell membrane staining and the percentage of tumor cells with complete, basolateral, or lateral membrane staining were evaluated. The nonneoplastic gastric mucosa consistently exhibited strong staining as a positive internal control, and the intensity was evaluated as either strong (3 +), moderate (2 +), weak (1 +), or none (0) (Fig. S1). The percentage of each stained tumor cell was assessed approximated to the nearest 10%. CLDN18.2 positivity was defined as moderate-to-strong (2 +/3 +) expression in ≥ 75% of the tumor cells in pretreatment biopsy specimens and/or surgically resected FFPE specimens. IHC analysis was conducted under the supervision of a pathologist (Y.M.) of the Department of Pathology, University of Osaka Hospital, who was blinded to the clinical data. Samples that could not be evaluated due to staining failure were substituted with alternative blocks.

### Intratumoral heterogeneity of CLDN18.2 in surgically resected specimens

**Fig. 1:**
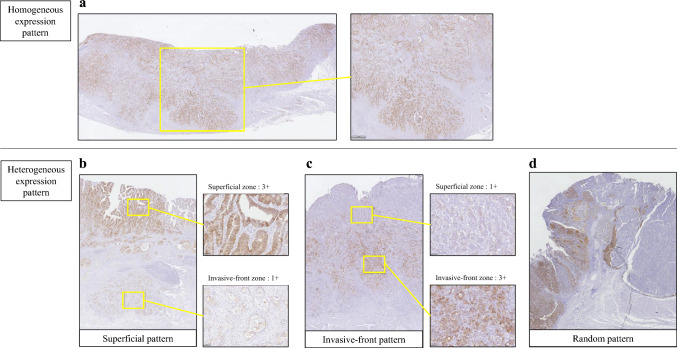
A representative image of the case showing CLDN18.2 intratumoral heterogeneity. **a** Homogeneous expression pattern: The tumor cells with a staining intensity of 3 + were evenly distributed in the tumor. **b** Superficial pattern: The tumor cells in the superficial zone showed a staining intensity of 3 +, whereas the tumor cells in the invasive-front zone showed a weak staining intensity of 1 +. **c** Invasive-front pattern: The tumor cells in the invasive-front zone showed a staining intensity of 3 +, whereas the tumor cells in the superficial zone showed a weak staining intensity of 1 +. **d** Random pattern: The distribution of various stained tumor cell expressions was patchy. *CLDN18.2 *Claudin-18 isoform-2

The intratumoral heterogeneity of CLDN18.2 was evaluated on surgically resected FFPE specimens. A homogeneous expression pattern was defined as expression in more than 90% of the area with a 2 +/3 + intensity, according to the previous studies [18, 19]. A heterogeneous expression pattern was defined as expression in 10%–90% of the area with a 2 +/3 + intensity, as we aimed to capture intratumoral variability even in cases with relatively low CLDN18.2 expression. The primary whole tumor area was subdivided into three zones from the mucosal surface toward the invasive margin: the superficial, central, and invasive-front zones [20]. CLDN18.2 expression was assessed separately within each zone, and the distributed pattern was classified as superficial, invasive-front, or random, as previously reported [18, 19]. Superficial pattern was defined as the highest expression predominantly located in the superficial zone, and invasive-front pattern as the most prominent expression in the invasive-front zone. Random pattern was assigned when neither criterion was met, characterized by a patchy distribution of staining with variable intensities without zonal predominance. Representative images of a homogeneous and three heterogeneous expression patterns are presented in Fig. 1.

### Statistical analysis

The associations between CLDN18.2 expression and clinicopathological characteristics were analyzed using the chi-squared test for categorical variables and the Mann–Whitney U test for continuous variables. Relapse-free survival (RFS) was defined as the interval from the date of surgery to the date of recurrence or death from any cause. Overall survival (OS) was defined as the interval from the date of surgery to the date of death from any cause. The survival rates were estimated using the Kaplan–Meier method and compared using the log-rank test. The hazard ratio (HR) for death in CLDN18.2-positive patients was estimated using a Cox proportional-hazards model. Multivariate Cox regression analysis was performed to adjust for potential confounding factors, including commonly used prognostic factors in gastric cancer. Concordance of CLDN18.2 positivity was evaluated using Cohen’s kappa coefficient (к) for biopsy–resection pairs, including pre- and post-neoadjuvant comparisons. The paired comparison of the percentage of CLDN18.2–positive tumor cells before and after chemotherapy was performed using the Wilcoxon signed-rank test. *P* < 0.05 was considered to indicate statistical significance. All statistical analyses were conducted using the SPSS software (version 29.0.2.0, IBM Corp., Armonk, NY, USA) and JMP software (Student Edition 18.2.1, SAS Institute, Cary, NC).

## Results

### CLDN18.2 expression status and clinicopathological characteristics

**Fig. 2:**
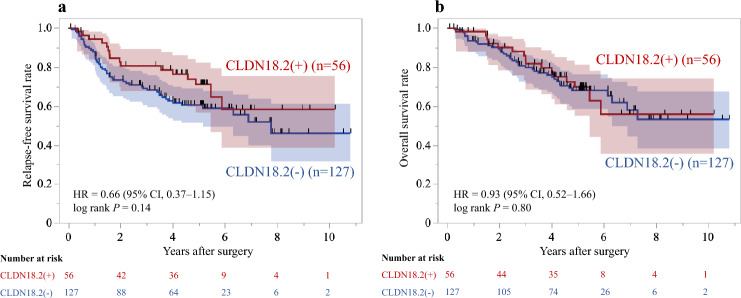
Kaplan–Meier relapse-free survival **a** and overall survival **b** according to the CLDN18.2 expression status in 183 patients who underwent upfront gastrectomy CLDN18.2 positivity was defined as moderate-to-strong (2 +/3 +) expression in ≥ 75% of the tumor cells in pretreatment specimens. *CLDN18.2 *Claudin-18 isoform-2,* HR *hazard ratio*, CI* confidence interval

IHC of CLDN18.2 showed that 56 (31%) of all 183 patients who underwent upfront gastrectomy were found to have CLDN18.2-positive expression. To assess potential temporal influences, we divided the entire cohort into two chronological periods; the first half (February 2013 – June 2016; n = 92) and the second half (June 2016 – December 2019; n = 91). The CLDN18.2 positivity rates were 28% (26/92) and 33% (30/91), respectively, with no significant difference between the periods (P = 0.49). The clinicopathological factors of all patients were compared by CLDN18.2 status (Table 1). CLDN18.2 positivity was observed in a higher proportion of men (*P* = 0.011). Tumors located in the upper body of the stomach were significantly more likely to be CLDN18.2-positive (*P* = 0.041). The other factors, including histological type and pathological stage, demonstrated no significant association with CLDN18.2 positivity. The RFS and OS of the 183 patients were analyzed to assess the prognostic impact of CLDN18.2 expression. Over a median follow-up of 4.2 years (interquartile range [IQR], 2.6–5.3), RFS events occurred in 16/56 CLDN-positive and 51/128 CLDN-negative patients, with no significant difference between groups (HR, 0.66; 95% confidence interval [CI], 0.37–1.15; log-rank P = 0.14) (Fig. 2a). For OS, during a median follow-up of 4.5 years (IQR, 1.6–5.2), 16 and 39 deaths occurred in the CLDN-positive and -negative groups, respectively, also showing no significant difference (HR, 0.93; 95% CI, 0.52–1.66; log-rank P = 0.80) (Fig. 2b). A Cox multivariate analysis of OS with CLDN18.2 status and seven clinicopathological factors (age, sex, location, histological type, pathological T status, pathological N status, adjuvant chemotherapy; ratio of events to covariates, 6.9) revealed that pathological T and N status (*P* = 0.003 and* P* = 0.004, respectively) were significant prognostic factors but that CLDN18.2 status was not (*P* = 0.58).

**Table 1:** Characteristics of 183 patients who underwent upfront gastrectomy

Characteristics	CLDN18.2 (+) (n = 56)	CLDN18.2 (−) (n = 127)	*P*
Age (years)			
Median (range)	74 (44–89)	71 (35–90)	0.19
Sex			
Male	47 (84%)	83 (63%)	0.011
Female	9 (16%)	44 (37%)	
Location			
Upper third	24 (43%)	35 (28%)	0.041
Middle/lower third	32 (57%)	92 (72%)	
Histological type			
Differentiated	32 (57%)	64 (50%)	0.40
Undifferentiated	24 (43%)	63 (50%)	
Pathological T status			
T2	24 (43%)	49 (39%)	0.70
T3	15 (27%)	42 (33%)	
T4	17 (30%)	36 (28%)	
Pathological N status			
N0	30 (54%)	58 (46%)	0.32
N1-3	26 (46%)	69 (54%)	
Lymphatic invasion			
Absent	12 (21%)	18 (14%)	0.22
Present	44 (79%)	109 (86%)	
Venous invasion			
Absent	28 (50%)	51 (40%)	0.22
Present	28 (50%)	76 (60%)	
Pathological stage			
I	19 (34%)	31 (25%)	0.41
II	19 (34%)	50 (39%)	
III	18 (32%)	46 (36%)	

*CLDN18.2 *Claudin-18 isoform-2

### CLDN18.2 intratumoral heterogeneity in surgically resected specimens

**Fig. 3:**
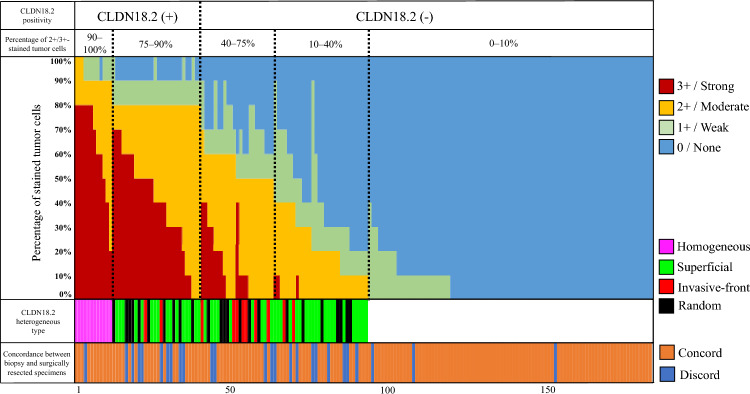
Visualization of the percentage of tumor cells in 183 surgically resected specimens per CLDN18.2 staining intensity. The percentage of each stained tumor cell in 183 surgically resected specimens without preoperative chemotherapy was assessed approximated to the nearest 10%. Claudin-18 isoform-2 (CLDN18.2) positivity was defined as moderate-to-strong (2 +/3 +) expression in ≥ 75% of the tumor cells. The percentage of 2 +/3 + -stained tumor cells was divided into 0%–10%, 10%–40%, 40%–75%, 75%–90%, and 90%–100% using dotted lines. Intratumoral heterogeneity was assessed in cases with ≥ 10% of tumor cells demonstrating moderate-to-strong (2 +/3 +) CLDN18.2 expression. Cases with one homogeneous and three heterogeneous expression patterns (superficial, invasive-front, and random pattern) are denoted by colors (purple, green, red, and black bars). The concordance of CLDN18.2 expression between the biopsy and surgically resected specimens is also denoted by colors (orange and blue bars)

To evaluate the intratumoral heterogeneity of CLDN18.2, the percentage of tumor cells per staining intensity in 183 surgically resected specimens was investigated **(**Fig. 3). The detailed IHC results for the percentage of tumor cells with moderate-to-strong CLDN18.2 expression in 183 tumors were as follows: 75%–100%, 40 (22%); 40%–75%, 30 (16%); 10%–40%, 23 (13%); and 0%–10%, 90 (49%). Of the 93 patients with 2 +/3 + expression in ≥ 10% of the tumor cells, 12 (13%) had homogeneous CLDN18.2 expression patterns and 81 (87%) had heterogeneous expression patterns, including superficial (n = 46), random (n = 24), and invasive-front (n = 11) patterns. The cohort was further stratified into two clinically defined groups: CLDN18.2-positive (≥ 75%) and negative (10–74%), and the superficial pattern was most common in both groups (Table S1).

### Comparison of CLDN18.2 expression between matched biopsy and surgically resected specimens

For each patient, the median number of biopsies from the primary tumor was 3 (range, 1–6). The positivity rate measured by specimen was 25% (46/183) for the biopsy specimen and 22% (40/183) for the surgically resected specimen. The overall biopsy–surgery concordance rate was 86% (157/183, к = 0.61, P < 0.001) (Table 2). The color map for the concordance between the pretreatment biopsy and surgically resected specimens for CLDN18.2 is presented in Fig. 3. Of the 12 patients with homogeneous expression patterns, 11 (92%) demonstrated concordance between the biopsy and surgically resected specimens. Meanwhile, only 59 (73%) of the 81 patients with heterogeneous expression patterns demonstrated concordance. As regards the three distributed patterns, the concordance rates were 65% (30/46, к = 0.31, P = 0.024), 79% (19/24, к = 0.55, P = 0.005), and 91% (10/11, к = 0.62, P = 0.026) for the superficial, random, and invasive-front patterns, respectively (Table 3). Moreover, of the 16 discordant cases with positive and negative CLDN18.2 expressions on the biopsy and surgically resected specimens, respectively, 75% exhibited the superficial pattern (**Table S2**). Of the 10 discordant cases with negative and positive expressions on the biopsy and surgically resected specimens, respectively, the superficial and random patterns were observed in four (40%) cases each (**Table S3**).

**Table 2:** Status of the CLDN18.2 expression between the matched preoperative biopsy and surgically resected specimens in 183 patients who underwent upfront gastrectomy

	Surgically resected specimen		Total
	CLDN18.2 (+)	CLDN18.2 (−)	
Biopsy specimen			
CLDN18.2 (+)	30	16	46
CLDN18.2 (−)	10	127	137
Total	40	143	183

*CLDN18.2* Claudin-18 isoform-2

**Table 3:** Status of CLDN18.2 expression between the matched preoperative biopsy and surgically resected specimens according to the heterogeneity patterns in 183 patients who underwent upfront gastrectomy

Heterogeneity patterns		Surgically resected specimen		Total
		CLDN18.2 (+)	CLDN18.2 (−)	
Homogeneous	Biopsy specimen			
	CLDN18.2 (+)	11	0	11
	CLDN18.2 (−)	1	0	1
	Total	12	0	12
		Concordance rate: 92%, к: N/A		
Superficial	Biopsy specimen			
	CLDN18.2 (+)	12	12	24
	CLDN18.2 (−)	4	18	22
	Total	16	30	46
		Concordance rate: 65%, к = 0.31 (P = 0.024)		
Invasive-front	Biopsy specimen			
	CLDN18.2 (+)	1	0	1
	CLDN18.2 (−)	1	9	10
	Total	2	9	11
		Concordance rate: 91%, к = 0.62 (P = 0.026)		
Random	Biopsy specimen			
	CLDN18.2 (+)	6	1	7
	CLDN18.2 (−)	4	13	17
	Total	10	14	24
		Concordance rate: 79%, к = 0.55 (P = 0.005)		
0–10% of CLDN18.2 positive cells	Biopsy specimen			
	CLDN18.2 (+)	0	3	3
	CLDN18.2 (−)	0	87	87
	Total	0	90	90
		Concordance rate: 97%, к: N/A		

Kappa values were interpreted based on the Landis and Koch criteria: slight (0.00–0.20), fair (0.21–0.40), moderate (0.41–0.60), substantial (0.61–0.80), and almost perfect agreement (0.81–1.00)

*N/A* not applicable,* CLDN18.2* Claudin-18 isoform-2

### Comparison of CLDN18.2 expression pre- and post-neoadjuvant chemotherapy

We evaluated CLDN18.2 expressions using pretreatment biopsy and surgically resected specimens from 38 patients who underwent neoadjuvant chemotherapy (DOS in 28 patients and SOX in 10) followed by gastrectomy. Patients with a histological response of Grade 3 were excluded from the entire study, in accordance with the eligibility criteria. Among the remaining 38 cases with a histological response Grade ≤ 2, 31 (82%) were classified as Grade ≤ 1b and 7 (18%) as Grade 2. The concordance rate of CLDN18.2 positivity between the biopsy specimens pre-neoadjuvant chemotherapy and surgically resected specimens post-neoadjuvant chemotherapy was 82% (31/38, к = 0.45, P < 0.001) (Table 4). Of the 11 patients who demonstrated CLDN18.2 positivity pre-neoadjuvant chemotherapy, only 4 were positive post-chemotherapy. Meanwhile, all the 27 patients who showed CLDN18.2 negativity pre-neoadjuvant chemotherapy exhibited negativity post-chemotherapy. Even when stratified by histological response, a similar trend was observed, with a concordance rate of 84% (26/31; κ = 0.53, P < 0.001) among patients with histological response Grade ≤ 1b (**Table S4**). Moreover, a paired comparison using the percentage of CLDN18.2–positive tumor cells (2 +/3 +) demonstrated a trend toward decreased expression following neoadjuvant chemotherapy (P = 0.061, **Figure S2**).

**Table 4:** Status of CLDN18.2 expression pre- and post-neoadjuvant chemotherapy in 38 patients who underwent neoadjuvant chemotherapy followed by gastrectomy

	Post-neoadjuvant chemotherapy		Total
	CLDN18.2 (+)	CLDN18.2 (−)	
Pre-neoadjuvant chemotherapy			
CLDN18.2 (+)	4	7	11
CLDN18.2 (−)	0	27	27
Total	4	34	38

*CLDN18.2* Claudin-18 isoform-2

## Discussion

In this study, a detailed analysis of the intratumoral heterogeneity of CLDN18.2 expression in surgically resected specimens was conducted. The results indicated that the superficial pattern was the most common distribution in the cases with heterogeneous expression patterns. We compared the CLDN18.2 expressions between the matched biopsy and surgically resected specimens and observed an overall concordance rate of 86%. The concordance rate markedly decreased in cases with heterogeneous expression patterns, particularly in those with superficial patterns. These results indicate that the intratumoral heterogeneity pattern of CLDN18.2 affects the evaluation of CLDN18.2 expression using clinical biopsy specimens.

Several studies have assessed CLDN18.2 expression in GC, but differences in antibodies and positivity thresholds likely account for the variability in reported expression rates [21]. We used the same antibodies and positive criteria as the landmark phase 3 trials, and the positive rate (31%) observed in our study was similar to those in previous ones, with rates of 24%–47% [8, 18, 22–26]. RFS and OS did not differ significantly between CLDN18.2-positive and -negative groups, and multivariate Cox analysis showed no survival impact of CLDN18.2 status in our cohort. This result is consistent with those of other studies, which indicated that CLDN18.2 may not be a prognostic biomarker [23, 24, 27, 28].

In previous studies that investigated heterogeneity CLDN18.2 expression patterns only in cases with 2 +/3 + expression in ≥ 75% of tumor cells, the random pattern was the most commonly observed [18, 19]. In contrast to previous studies, our cohort included cases with 2 +/3 + expression in ≥ 10% of tumor cells to investigate CLDN18.2 heterogeneity across a broader range of positivity, allowing assessment of variability even in tumors with relatively low expression, and revealed that the superficial pattern was the most frequently encountered distribution. This pattern reflects reduced CLDN18.2 expression at the invasive front of the GC, suggesting an association with tumor progression and the subsequent metastatic events, similar to other cancers [29–32]. These findings are consistent with those of a previous IHC study involving 22 surgically resected GC specimens from CLDN18.2-negative cases; this IHC study employed a methodology comparable to ours [20]. The predominance of the superficial pattern may affect the reliability of biopsy-based assessment because biopsy specimens typically sample only the superficial mucosal layer. In such cases, patients may be diagnosed as CLDN18.2-positive based on biopsy, despite not meeting the ≥ 75% cutoff when the entire tumor is evaluated.

Although several studies have investigated the heterogeneity of CLDN18.2 expression, none have evaluated its impact on the assessment of biopsy specimens. We demonstrated that the superficial pattern may increase the positive rate for CLDN18.2 in biopsy specimens compared with that in surgically resected specimens. However, the difference in the positive rates between the specimens in our cohort was only 3% (25% vs. 22%). As most samples in the SPOTLIGHT and GLOW trials were biopsies (81% vs. 19% resected specimens) [22], biopsy specimens are likely sufficient to evaluate CLDN18.2 expression. In addition, in our cohort, there was one case with a homogeneous expression pattern in which the biopsy was CLDN18.2-negative, and three cases with 0–10% CLDN18.2-positive cells in the surgical specimens in which the biopsies were positive. This marked discrepancy may suggest that the biopsy specimens obtained from tumor regions with entirely different characteristics compared to those assessed in the resected specimens. For CLDN18.2 testing, multiple biopsies (six or more biopsies) from different tumor regions are recommended to account for intratumoral heterogeneity as previously reported [23]. To reduce the impact of spatial heterogeneity, biopsies including deeper tumor regions are preferable, although routine endoscopic sampling is limited. When biopsy results are uncertain, surgically resected specimens can be used as a reference.

In this study, we investigated the changes in CLDN18.2 expression pre- and post-chemotherapy without zolbetuximab, and found the concordance rate was 82%. In a retrospective Japanese study of unresectable or metastatic GC, biopsy concordance before and after first-line chemotherapy was 75% (13/17) [24]. Meanwhile, Shitara et al. reported a 61% (22/36) concordance of CLDN18.2 positivity between archival and baseline tumor samples after treatment in unresectable or metastatic GC [22]. In our cohort, 7 of the 11 CLDN18.2-positive cases pre-neoadjuvant chemotherapy became negative post-chemotherapy, whereas no cases demonstrated a negative-to-positive change. This trend is consistent with those in previous studies [8, 22, 24]. The shift in the CLDN18.2 status from positive to negative post-chemotherapy could be attributed to the direct effect of chemotherapy on the CLDN18.2 expression itself as well as the impact of chemotherapy-induced tumor reduction on the evaluation of CLDN18.2-stained cells. As chemotherapy prior to CLDN18.2-target therapy may induce a shift in CLDN18.2 expression from positive to negative, it would be preferable to use the most recently collected samples which are considered to best reflect the current tumor status for companion diagnosis, particularly when the CLDN18.2-target therapy is considered in later treatment lines. However, if CLDN18.2 negativity has already been confirmed in previously resected specimens, reassessing its expression using a recent post-chemotherapy specimen would not be needed.

This study has several limitations that need to be acknowledged. First, it followed a retrospective design and was conducted in a single institution, which may limit the generalizability of our findings. However, we considered that the methods for specimen preservation and evaluation were the same; thus, there was some advantage as regards consistency in the assessment of CLDN18.2 expression. The fact that CLDN18.2 positivity rates were similar between older and newer cases suggests that tissue preservation at our institution was not a limiting factor. Second, we assessed CLDN18.2 in multiple biopsies but only one section per resected specimen, which may underestimate expression in deeper tumor regions. However, this approach reflects routine pathological practice and ensured consistency across cases. Future studies incorporating multi-section analyses may provide a more comprehensive assessment of intratumoral heterogeneity. Third, in the comparison between biopsy and surgically resected specimens and pre- and post-chemotherapy, the small number of discordant cases made it difficult to analyze more detailed characteristics. Nevertheless, to the best of our knowledge, this is the largest study to date to specifically address the issue of concordance. Lastly, the present study did not assess the inter-institutional or inter-observer variability in the evaluation of CLDN18.2 expression. In previous phase 3 trials, CLDN18.2 expression was reported to exhibit a bimodal distribution, with tumors clustering at either 0% or ≥ 75% positivity [22], suggesting that inter-institutional or inter-observer variability may not be a major concern. However, whether such variability truly has minimal impact remains to be fully evaluated, and future perspectives should include the potential role of digital pathology or AI-assisted scoring to enhance consistency and standardization.

In conclusion, we comprehensively investigate the intratumoral heterogeneity of CLDN18.2 in resectable GC, and demonstrated that the common heterogeneous type was the superficial pattern, followed by the random pattern, which has the potential to decrease the concordance rate between the biopsy and surgically resected specimens. Chemotherapy may alter CLDN18.2 expression from positive to negative. To select the best treatment for the patient, accurate determination of CLDN18.2 positivity is imperative. The study will provide better understanding of the intratumoral heterogeneity of CLDN18.2.

## Supplementary Information

Below is the link to the electronic supplementary material.

Supplementary file1 (PDF 889 KB)

## Data Availability

The data set generated and/or analyzed during the current study are available from the corresponding author on reasonable request.

## References

[1] Bray F, Laversanne M, Sung H et al (2024) Global cancer statistics 2022: GLOBOCAN estimates of incidence and mortality worldwide for 36 cancers in 185 countries. CA Cancer J Clin 74:229–263 38572751 10.3322/caac.21834

[2] Tokunaga M, Kurokawa Y, Fukagawa T et al (2023) Neoadjuvant chemotherapy for locally advanced gastric cancer in Japan: consensus meeting at the 77th general meeting of the Japanese Society of Gastroenterological Surgery. Ann Gastroenterol Surg 7:856–862 37927916 10.1002/ags3.12717 PMC10623975

[3] Higashi T, Kurokawa Y (2024) Incidence, mortality, survival, and treatment statistics of cancers in digestive organs-Japanese cancer statistics 2024. Ann Gastroenterol Surg 8:958–965 39502737 10.1002/ags3.12835 PMC11533006

[4] Kurokawa Y, Doki Y, Mizusawa J et al (2018) Bursectomy versus omentectomy alone for resectable gastric cancer (JCOG1001): a phase 3, open-label, randomised controlled trial. Lancet Gastroenterol Hepatol 3:460–468 29709558 10.1016/S2468-1253(18)30090-6

[5] Yanagimoto Y, Kurokawa Y, Doki Y (2023) Essential updates 2021/2022: perioperative and surgical treatments for gastric and esophagogastric junction cancer. Ann Gastroenterol Surg 7:698–708 37663969 10.1002/ags3.12711 PMC10472390

[6] Yao F, Kausalya JP, Sia YY et al (2015) Recurrent Fusion Genes in Gastric Cancer: CLDN18-ARHGAP26 Induces Loss of Epithelial Integrity. Cell Rep 12:272–285 26146084 10.1016/j.celrep.2015.06.020

[7] Sahin U, Koslowski M, Dhaene K et al (2008) Claudin-18 splice variant 2 is a pan-cancer target suitable for therapeutic antibody development. Clin Cancer Res 14:7624–7634 19047087 10.1158/1078-0432.CCR-08-1547

[8] Waters R, Sewastjanow-Silva M, Yamashita K et al (2024) Retrospective study of claudin 18 isoform 2 prevalence and prognostic association in gastric and gastroesophageal junction adenocarcinoma. JCO Precis Oncol 8:e2300543 38781542 10.1200/PO.23.00543 PMC11371102

[9] Jia K, Chen Y, Sun Y et al (2022) Multiplex immunohistochemistry defines the tumor immune microenvironment and immunotherapeutic outcome in CLDN18.2-positive gastric cancer. BMC Med 20:223 35811317 10.1186/s12916-022-02421-1 PMC9272556

[10] Shah MA, Shitara K, Ajani JA et al (2023) Zolbetuximab plus CAPOX in CLDN18.2-positive gastric or gastroesophageal junction adenocarcinoma: the randomized, phase 3 GLOW trial. Nat Med 29:2133–2141 37524953 10.1038/s41591-023-02465-7 PMC10427418

[11] Shitara K, Lordick F, Bang Y-J et al (2023) Zolbetuximab plus mFOLFOX6 in patients with CLDN18.2-positive, HER2-negative, untreated, locally advanced unresectable or metastatic gastric or gastro-oesophageal junction adenocarcinoma (SPOTLIGHT): a multicentre, randomised, double-blind, phase 3 trial. Lancet 401:1655–1668 37068504 10.1016/S0140-6736(23)00620-7

[12] Kurokawa Y, Matsuura N, Kimura Y et al (2015) Multicenter large-scale study of prognostic impact of HER2 expression in patients with resectable gastric cancer. Gastric Cancer 18:691–697 25224659 10.1007/s10120-014-0430-7

[13] Grillo F, Fassan M, Sarocchi F et al (2016) HER2 heterogeneity in gastric/gastroesophageal cancers: From benchside to practice. World J Gastroenterol 22:5879–5887 27468182 10.3748/wjg.v22.i26.5879 PMC4948273

[14] Mittendorf EA, Wu Y, Scaltriti M et al (2009) Loss of HER2 amplification following trastuzumab-based neoadjuvant systemic therapy and survival outcomes. Clin Cancer Res 15:7381–7388 19920100 10.1158/1078-0432.CCR-09-1735 PMC2788123

[15] Shu S, Iimori M, Nakanishi R et al (2018) Changes in HER2 expression and amplification status following preoperative chemotherapy for gastric cancer. In Vivo 32:1491–1498 30348707 10.21873/invivo.11405 PMC6365720

[16] Kijima T, Arigami T, Uenosono Y et al (2020) Comparison of HER2 status before and after trastuzumab-based chemotherapy in patients with advanced gastric cancer. Anticancer Res 40:75–80 31892554 10.21873/anticanres.13927

[17] (2017) Japanese Classification Gastric Carcinoma. Kanehara Shuppan, Tokyo

[18] Choi E, Shin J, Ryu M-H et al (2024) Heterogeneity of claudin 18.2 expression in metastatic gastric cancer. Sci Rep 14:17648 39085339 10.1038/s41598-024-68411-w PMC11291723

[19] Kim H-D, Choi E, Shin J et al (2023) Clinicopathologic features and prognostic value of claudin 18.2 overexpression in patients with resectable gastric cancer. Sci Rep 13:20047 37973935 10.1038/s41598-023-47178-6 PMC10654731

[20] Ogawa H, Abe H, Yagi K et al (2024) Claudin-18 status and its correlation with HER2 and PD-L1 expression in gastric cancer with peritoneal dissemination. Gastric Cancer 27:802–810 38724721 10.1007/s10120-024-01505-6 PMC11193835

[21] Angerilli V, Ghelardi F, Nappo F et al (2024) Claudin-18.2 testing and its impact in the therapeutic management of patients with gastric and gastroesophageal adenocarcinomas: a literature review with expert opinion. Pathol Res Pract 254:155145 38277741 10.1016/j.prp.2024.155145

[22] Shitara K, Xu R-H, Ajani JA et al (2024) Global prevalence of claudin 18 isoform 2 in tumors of patients with locally advanced unresectable or metastatic gastric or gastroesophageal junction adenocarcinoma. Gastric Cancer 27:1058–1068 38954176 10.1007/s10120-024-01518-1 PMC11335819

[23] Pellino A, Brignola S, Riello E et al (2021) Association of CLDN18 protein expression with clinicopathological features and prognosis in advanced gastric and gastroesophageal junction adenocarcinomas. J Pers Med. 10.3390/jpm11111095 34834447 10.3390/jpm11111095 PMC8624955

[24] Kubota Y, Kawazoe A, Mishima S et al (2023) Comprehensive clinical and molecular characterization of claudin 18. 2 expression in advanced gastric or gastroesophageal junction cancer. ESMO Open 8(1):100762 36610262 10.1016/j.esmoop.2022.100762 PMC10024138

[25] Kim T-Y, Kwak Y, Nam SK et al (2024) Clinicopathological analysis of claudin 18.2 focusing on intratumoral heterogeneity and survival in patients with metastatic or unresectable gastric cancer. ESMO Open 9:104000 39615405 10.1016/j.esmoop.2024.104000 PMC11648117

[26] Okazaki U, Nakayama I, Sakamoto N et al (2024) Clinical implementation of simultaneous multiple biomarkers testing for metastatic or recurrent gastroesophageal adenocarcinoma: a single-institutional experience. ESMO Gastrointestinal Oncology 5:100086 41647584 10.1016/j.esmogo.2024.100086 PMC12836672

[27] Arnold A, Daum S, von Winterfeld M et al (2020) Prognostic impact of Claudin 18.2 in gastric and esophageal adenocarcinomas. Clin Transl Oncol 22:2357–2363 32488802 10.1007/s12094-020-02380-0 PMC7577914

[28] Coati I, Lotz G, Fanelli GN et al (2019) Claudin-18 expression in oesophagogastric adenocarcinomas: a tissue microarray study of 523 molecularly profiled cases. Br J Cancer 121:257–263 31235864 10.1038/s41416-019-0508-4 PMC6738069

[29] Matsuda Y, Semba S, Ueda J et al (2007) Gastric and intestinal claudin expression at the invasive front of gastric carcinoma. Cancer Sci 98:1014–1019 17459057 10.1111/j.1349-7006.2007.00490.x PMC11159341

[30] Oshima T, Shan J, Okugawa T et al (2013) Down-regulation of claudin-18 is associated with the proliferative and invasive potential of gastric cancer at the invasive front. PLoS ONE 8:e74757 24073219 10.1371/journal.pone.0074757 PMC3779237

[31] Ueda J, Semba S, Chiba H et al (2007) Heterogeneous expression of claudin-4 in human colorectal cancer: decreased claudin-4 expression at the invasive front correlates cancer invasion and metastasis. Pathobiology 74:32–41 17496431 10.1159/000101049

[32] Usami Y, Chiba H, Nakayama F et al (2006) Reduced expression of claudin-7 correlates with invasion and metastasis in squamous cell carcinoma of the esophagus. Hum Pathol 37:569–577 16647955 10.1016/j.humpath.2005.12.018

