# Supplementary material for: Intratumoral heterogeneity and chemotherapy-induced alteration of CLDN18.2 expression in resectable gastric cancer
Source: Int J Clin Oncol. 2026 Jan 28;31(4):621–9. doi: 10.1007/s10147-026-02972-w (PMC13018064; doi:10.1007/s10147-026-02972-w)

International Journal of Clinical Oncology

Original article

Intratumoral heterogeneity and chemotherapy-induced alteration of CLDN18.2 expression in resectable gastric cancer

Shinnosuke Nagano[1], Yukinori Kurokawa[1*], Takaomi Hagi[1], Yuichi Motoyama[2], Takuro Saito[1], Tsuyoshi Takahashi[1], Kota Momose[1], Kotaro Yamashita[1], Koji Tanaka[1], Tomoki Makino[1], Kiyokazu Nakajima[1], Eiichi Morii[2], Hidetoshi Eguchi[1], Yuichiro Doki[1]

[1] Department of Gastroenterological Surgery, Graduate School of Medicine, The University of Osaka, 2-2 Yamadaoka, Suita City, Osaka 565-0871, Japan

[2] Department of Pathology, Graduate School of Medicine, The University of Osaka, 2-2 Yamadaoka, Suita City, Osaka 565-0871, Japan

*Corresponding author:

Yukinori Kurokawa, MD, PhD

Department of Gastroenterological Surgery, Graduate School of Medicine, The University of Osaka, 2-2 Yamadaoka, Suita City, Osaka 565-0871, Japan

Tel: +81- (0) 6-6879-3251

Fax: +81-(0) 6-6879-3259

E-mail: ykurokawa@gesurg.med.osaka-u.ac.jp

Table S1. Heterogeneity patterns of CLDN18.2 expression in 93 patients with ≥10% 2+/3+ expression: overall and stratified by expression level (≥75% vs 10–74%)

	Overall (n = 93)	Percentage of 2+/3+ stained tumor cells	
		≥75% (n = 40)	10–74% (n = 53)
Homogeneous pattern	12 (13%)	12 (30%)	0 (0%)
Superficial pattern	46 (49%)	16 (40%)	30 (57%)
Random pattern	24 (26%)	10 (25%)	14 (26%)
Invasive-front pattern	11 (12%)	2 (5%)	9 (17%)

CLDN18.2, Claudin-18 isoform-2

Table S2. Summary of 16 cases with CLDN18.2-positive biopsy specimens and CLDN18.2-negative surgically resected specimens

No.	Number of biopsies	Surgically resected specimen		
		Histological type	Percentage of 2+/3+-stained tumor cells	Heterogeneous pattern
1	2	Differentiated	60%	Superficial
2	3	Differentiated	60%	Superficial
3	3	Differentiated	50%	Superficial
4	3	Undifferentiated	50%	Superficial
5	2	Undifferentiated	40%	Superficial
6	1	Undifferentiated	40%	Superficial
7	5	Differentiated	30%	Superficial
8	3	Undifferentiated	20%	Superficial
9	3	Differentiated	20%	Superficial
10	2	Differentiated	20%	Superficial
11	4	Undifferentiated	10%	Superficial
12	3	Differentiated	10%	Superficial
13	4	Differentiated	10%	Random
14	3	Differentiated	0%	-
15	4	Differentiated	0%	-
16	5	Undifferentiated	0%	-

CLDN18.2, Claudin-18 isoform-2

Table S3. Summary of 10 cases with CLDN18.2-negative biopsy specimens and CLDN18.2-positive surgically resected specimens

No.	Number of biopsies	Surgically resected specimen		
		Histological type	Percentage of 2+/3+-stained tumor cells	Heterogeneous pattern
1	2	Differentiated	80%	Superficial
2	3	Differentiated	80%	Superficial
3	4	Undifferentiated	80%	Superficial
4	4	Differentiated	80%	Superficial
5	4	Differentiated	80%	Random
6	3	Differentiated	80%	Random
7	3	Differentiated	80%	Random
8	4	Differentiated	80%	Random
9	3	Differentiated	80%	Invasive front
10	3	Differentiated	90%	Homogeneous

CLDN18.2, Claudin-18 isoform-2

Table S4. Status of CLDN18.2 expression pre- and post-neoadjuvant chemotherapy according to histological response Grade ≤1b (a) and Grade ≥2 (b)

(a)

		Post-neoadjuvant chemotherapy		Total
		CLDN18.2 (+)	CLDN18.2 (-)	
Pre-neoadjuvant chemotherapy	CLDN18.2 (+)	4	5	9
	CLDN18.2 (-)	0	22	22
	Total	4	27	31

(b)

		Post-neoadjuvant chemotherapy		Total
		CLDN18.2 (+)	CLDN18.2 (-)	
Pre-neoadjuvant chemotherapy	CLDN18.2 (+)	0	2	2
	CLDN18.2 (-)	0	5	5
	Total	0	7	7

CLDN18.2, Claudin-18 isoform-2

Figure S1. CLDN18.2 expression in gastric cancer cells

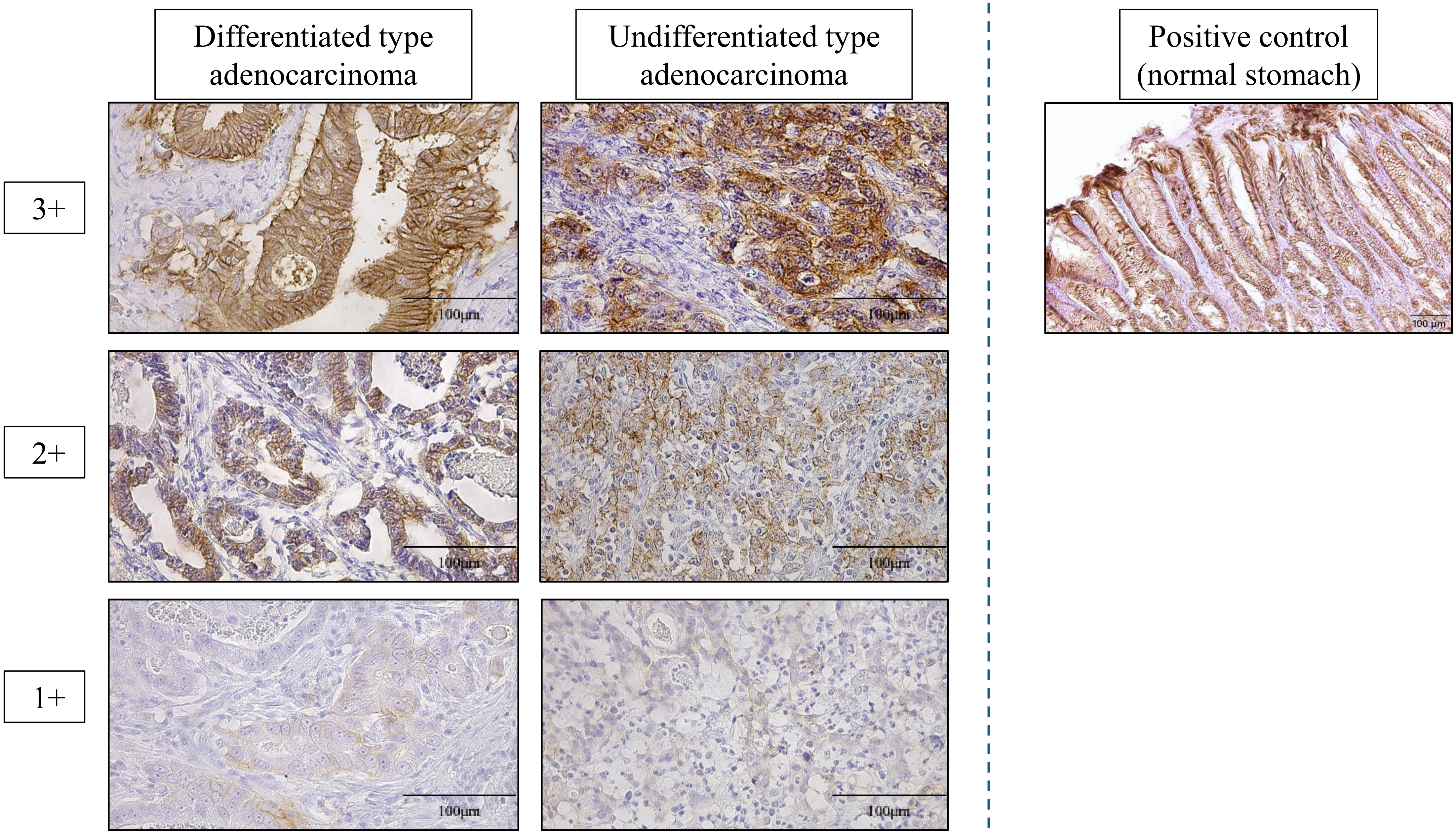

Figure S2. Comparison of the percentage of CLDN18.2–positive tumor cells (2+/3+) pre- and post-neoadjuvant chemotherapy in 38 patients who underwent neoadjuvant chemotherapy followed by gastrectomy

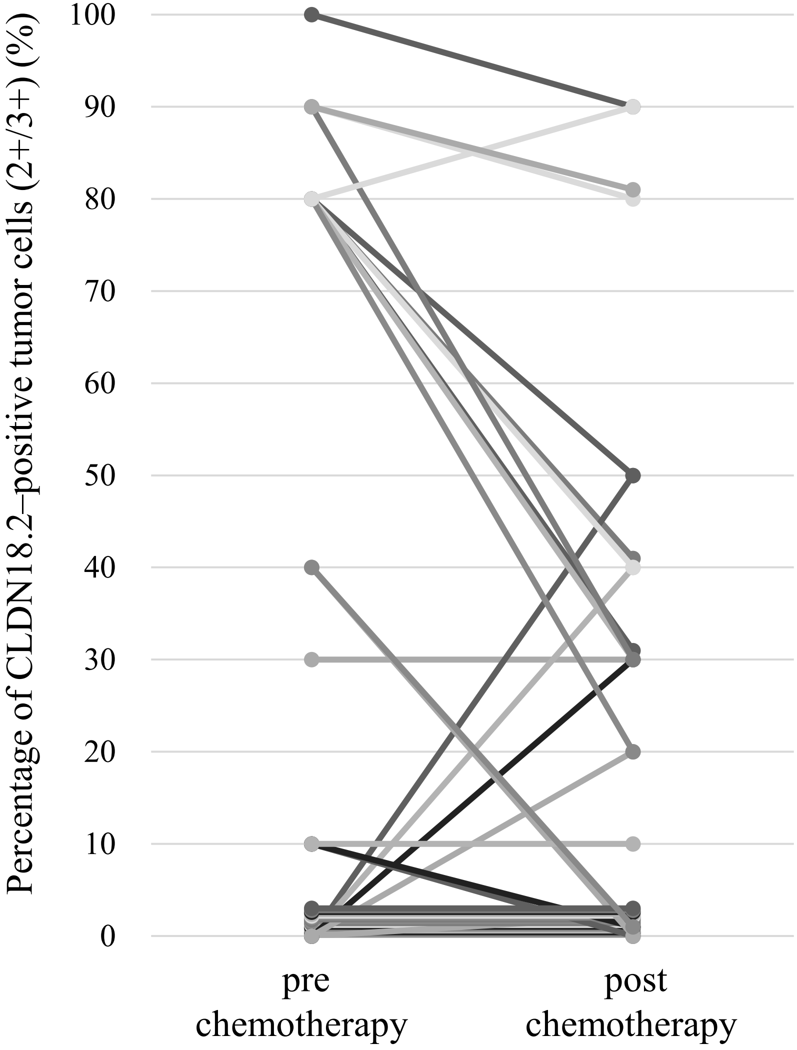

Supplement: Supplementary file 1 — Supplementary file1 (PDF 889 KB) [file 10147_2026_2972_MOESM1_ESM.pdf]
